# Simultaneous Determination of Multiple Classes of Hydrophilic and Lipophilic Components in Shuang-Huang-Lian Oral Liquid Formulations by UPLC-Triple Quadrupole Linear Ion Trap Mass Spectrometry

**DOI:** 10.3390/molecules22122057

**Published:** 2017-11-24

**Authors:** Jun Liang, Hui-Min Sun, Tian-Long Wang

**Affiliations:** Key Laboratory of Chinese MateriaMedica, Heilongjiang University of Chinese Medicine, Ministry of Education, Harbin 150040, China; hhuiminsun@163.com (H.-M.S.); tl890322@163.com (T.-L.W.)

**Keywords:** UPLC-MS/MS, Shuang-Huang-Lian, multi-ingredient quantitative analysis, quality control, principal component analysis

## Abstract

The Shuang-Huang-Lian (SHL) oral liquid is a combined herbal prescription used in the treatment of acute upper respiratory tract infection, acute bronchitis and pneumonia. Multiple constituents are considered to be responsible for the therapeutic effects of SHL. However, the quantitation of the multi-components from multiple classes is still unsatisfactory because of the high complexity of constituents in SHL. In this study, an accurate, rapid, and specific UPLC-MS/MS method was established for simultaneous quantification of 18 compounds from multiple classes in SHL oral liquid formulations. Chromatographic separation was performed on a HSS T3 (1.8 μm, 2.1 mm × 100 mm) column, using a gradient mobile phase system of 0.1% formic acid in acetonitrile and 0.1% formic acid in water at a flow rate of 0.2 mL·min^−1^; the run time was 23 min. The MS was operated in negative electrospray ionization (ESI^−^) for analysis of 18 compounds using multiple reaction monitoring (MRM) mode. UPLC-ESI^−^-MRM-MS/MS method showed good linear relationships (*R*^2^ > 0.999), repeatability (RSD < 3%), precisions (RSD < 3%) and recovery (84.03–101.62%). The validated method was successfully used to determine multiple classes of hydrophilic and lipophilic components in the SHL oral liquids. Finally, principal component analysis (PCA) was used to classify and differentiate SHL oral liquid samples attributed to different manufacturers of China. The proposed UPLC-ESI^−^-MRM-MS/MS coupled with PCA has been elucidated to be a simple and reliable method for quality evaluation of SHL oral liquids.

## 1. Introduction

Shuang-Huang-Lian (SHL) is a famous Chinese formula prepared from three Traditional Chinese Medicines (TCMs) including Lonicera Japonica (Jinyinhua), Radix Scutellariae (Huangqin) and Fructus Forsythiae (Lianqiao). SHL oral liquid has been widely applied to be effective clinical therapeutics for the treatment of acute upper respiratory, tract infection, acute bronchitis and pneumonia [1,2]. Flavonoids, lignans and phenylpropanoids have been confirmed to be the main and effective components in SHL oral liquid [1,3]. These ingredients were proven to be responsible for the various biological activities of this Chinese formula [1,2,3,4]. Many of these natural products displayed a wide range of biological activities such as antibacterial, antioxidant, anti-inflammatory activities. So quantification of these compounds would be of great significance to guarantee good quality of this formula of SHL.

Although quantitative analysis of single and a few compounds in this formula has been reported by capillary electrophoresis with electrochemical detection (CE-ECD) [5], high performance liquid chromatography with diode array detection (HPLC-DAD) [6], HPLC with mass spectrometry (HPLC-MS) [7], HPLC with evaporative light scattering detection (HPLC-ELSD) [8], and HPLC-DAD-ECD [9], a simultaneous analysis of flavonoids, lignans and phenylpropanoids in this prescription is still missing through UPLC-ESI-MS/MS based on multiple reaction monitoring (MRM) for speedy quality control.

UPLC is based on available reverse phase chromatographic media with a 1.7 μm particle size, together with a liquid system that can be operate such columns at much higher pressures. In comparison with common HPLC, UPLC offers many advantages including higher separation efficiency, shorter analysis time and less solvent consumption. Furthermore, UPLC hyphenated MS technique offers the possibility to obtain a more comprehensive chemical profiles and quantization by utilizing different ion modes and high sensitivity [10,11].

In this study, a simple, accurate and sensitive method based on UPLC-ESI^−^-MRM-MS/MS was developed for simultaneous determination of multiple hydrophilic and lipophilic components from multiple classes attributed to two quinic acids (**1** and **11**), one phenylpropionic acid (**2**), three phenylethanoid glycosides (**3**, **4** and **7**), eight flavonoids (**5**, **6**, **8**, **9**, **12**, **13**, **17** and **18**), four lignans (**10** and **14**–**16**). These compounds were reported in SHL oral liquids, including chlorogenic acid (**1**), caffeic acid (**2**), lianqiaoxinside A (**3**), forsythiaside B (**4**), rutin (**5**), hyperoside (**6**), forsythiaside A (**7**), cynaroside (**8**), scutellarin (**9**), (+)-pinoresinol-β-d-glucoside (**10**), isochlorogenic acid A (**11**), baicalein (**12**), baicalin (**13**), phillyrin (**14**), phillygenin (**15**), arctiin (**16**), quercetin (**17**) and luteolin (**18**). The current study provided a further refinement of the methods for the quality control of this traditional prescription.

## 2. Results and Discussion

### 2.1. Optimization of UPLC-MS/MS Conditions

Three reversed-phase chromatographic columns, including ACQUITY UPLC HSS T3 (1.8 μm, 2.1 mm × 100 mm), ACQUITY UPLC BEH C_18_ (1.7 μm, 2.1 mm × 100 mm) and Cortecs UPLC C_18_ (1.6 μm, 2.1 mm × 100 mm) were tested with the same sample solution. The results showed that the HSS T3 column displayed acceptable separation capacity. ACN/H_2_O system provided the best performance through the optimization of different mobile phases (MeOH/H_2_O, ACN/H_2_O and ACN/MeOH/H_2_O). Several different modifiers were investigated (none, formic acid, and ammonium formate), and the results showed that formic acid provided the best peak shape. Additional UPLC conditions were optimized by varying column temperatures (25, 30, 35 and 40 °C), and flow rates (0.2, 0.3 and 0.40 mL/min). The optimized UPLC conditions provided the highest selectivity and resolution. These were: ACQUITY UPLC HSS T3 column at 35 °C, 0.1% formic acid in water (A) and 0.1% formic acid in acetonitrile (B) mobile phase gradient at a flow rate of 0.20 mL/min.

**Figure 1:**
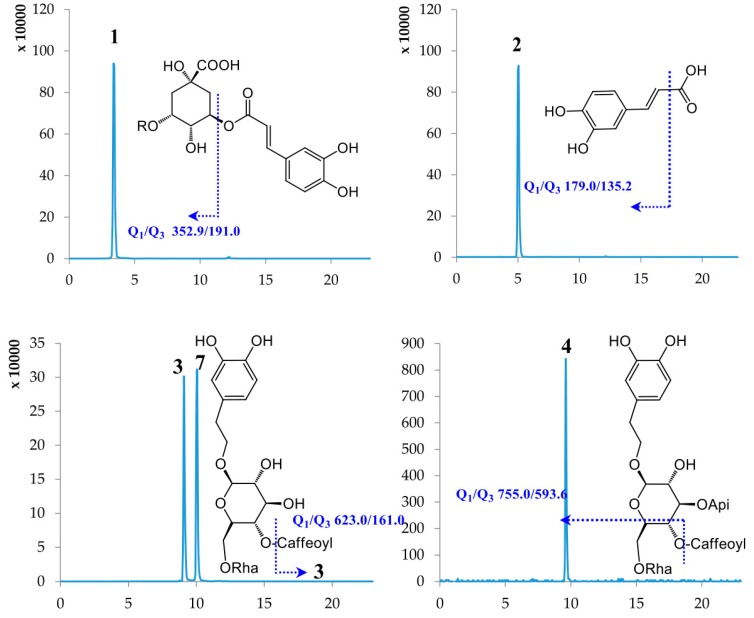

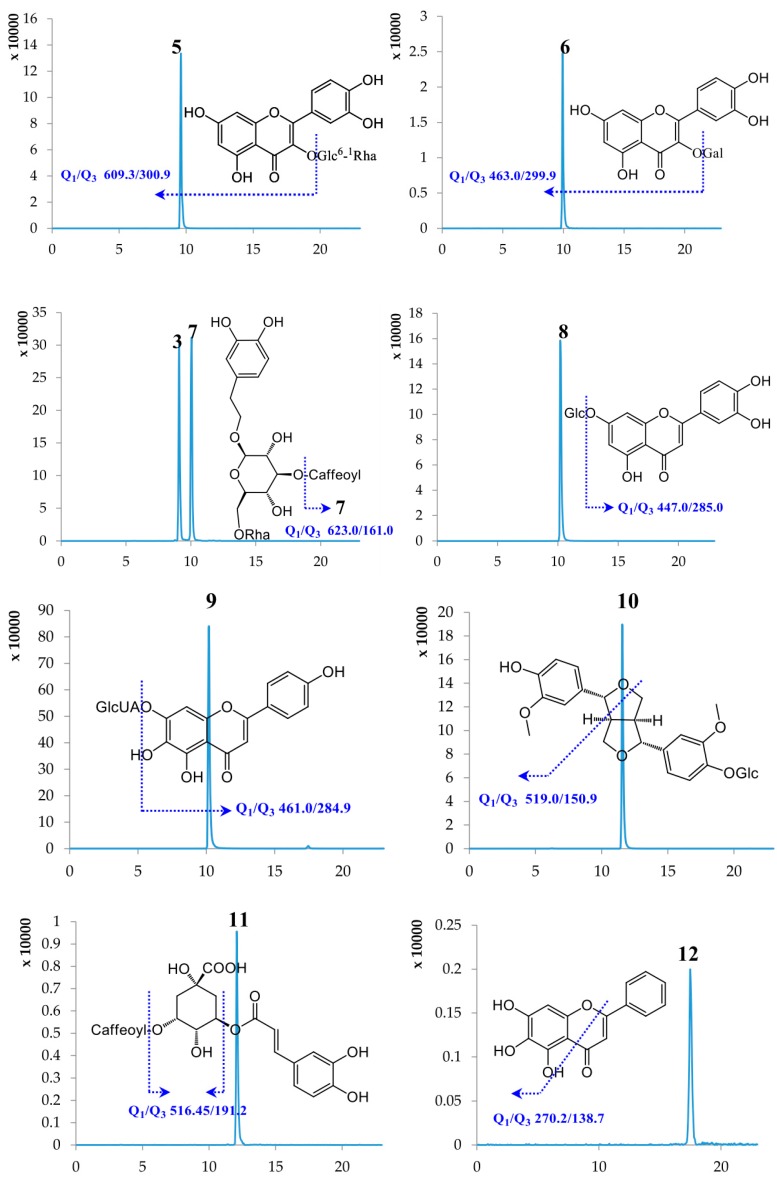

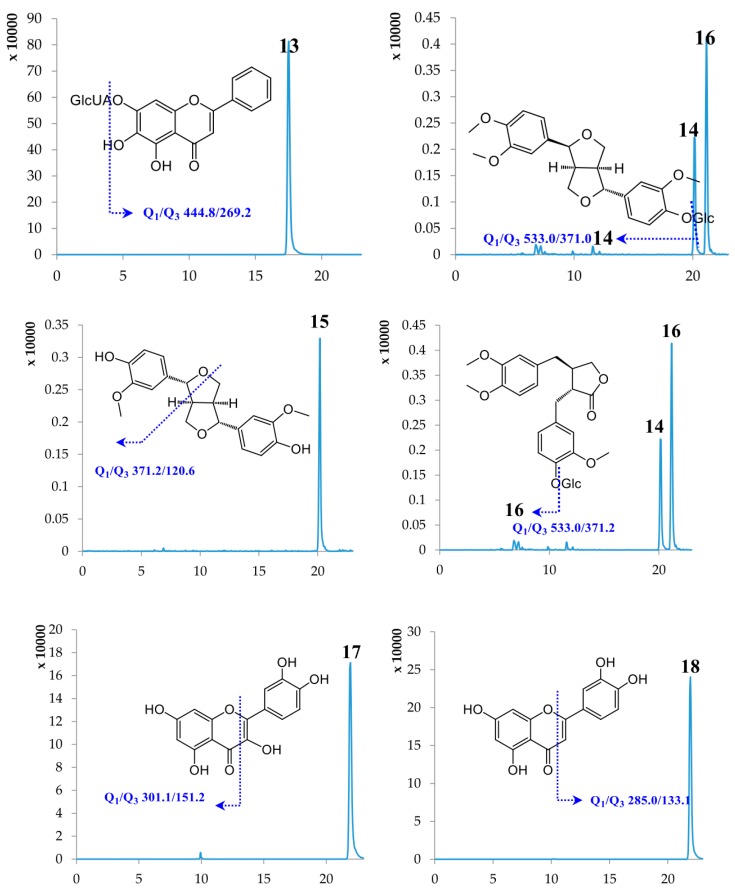
MRM chromatograms and their major fragmentations of reference standards **1**–**18**.

Under the chosen chromatographic conditions, compounds (**1**–**18**) showed to be present of the more intensive [M − H]^−^ ions in the negative ion mode than those of [M + H]^+^ or [M + Na]^+^ in the positive mode. Thus, a 4000 QTRAP UPLC-MS/MS system equipped with ESI interface in negative mode was used for detection in MRM mode. As shown in Figure 1, reference standards (**1**–**18**) showed good peak shapes and excellent resolutions. The main MS parameters including declustering potential (DP), collision energy (CE) were acquired and summarized in Table 1.

The major fragmentation pathways of **1**–**18** were also clarified in Figure 1. Compounds **1** and **11** afforded major product ion at *m*/*z* 191.0 due to the preferential cleavage of ester glycosidic bonds. A neutral loss of CO_2_ was readily observed for compound **2** to produce typical product ion at *m*/*z* 135.2. The cleavage of ester glycosidic bonds was observed for compounds **3**, **4** and **7** due to the present of the caffeoyl moieties. The compounds **5**, **6**, **8**, **9**, **13**, **14** and **16** underwent dissociation of monosaccharide glycosidic bonds to give the corresponding product ions at *m*/*z* 300.9, 299.9, 285.0, 284.9, 371.0 and 371.2, respectively. The characteristic product ion at *m*/*z* 150.9 for compound **10** was formed by cross-ring cleavages of tetrahydrofuran rings. Three flavonoids **12**, **17** and **18** experienced the retro-Diels-Alder fragmentation reaction of C-ring opening and B-ring cleavage to afford the corresponding product ions at *m*/*z* 138.7, 151.2 and 133.1, respectively. Therefore, the MRM transition used those intact deprotonated *m*/*z* values of precursors (Q_1_) and major fragments as product ions (Q_3_) for accurate detection of these compounds **1**–**18**.

### 2.2. Method Validation

The results of calibration were summarized in Table 2 and good correlations were found between the peak area (*y*) and concentration of tested compounds (*x*) (*r* > 0.999) within test ranges. The limit of detections (LODs) and the limit of quantifications (LOQs) for all standard analytes were in the range of 2.44–78.13 ng/mL and 4.88–156.25 ng/mL, respectively, indicating that this method is sensitive for the quantitative determination of major components in SHL oral liquid samples.

Repeatability of this method was obtained by analyzing six different samples using the same preparation procedure. RSD values of component content and retention time of these 18 compounds were all less than 3.0%, which satisfied the criteria of quantitative analysis.

Intra-day and inter-day variability was used to evaluate precision. Six sample solutions respectively prepared as described above and mixed standard solutions of eighteen compounds at low, medium and high concentrations on 1 day (*n* = 6) and on three consecutive days, were analyzed, respectively. The results indicated that the mean intra-day and inter-day RSD were less than 3.0%. Results from determination of intra-day and inter-day precision (as RSD) are shown in Table S1.

For the stability test, retention time and peak area of eighteen compounds in sample solution were analyzed in 0, 2, 4, 8, 16, 32 and 48 h. RSD values of the retention time and peak area of eighteen compounds were less than 0.5% and 3.0%, respectively. These results suggested that it was feasible to analyze samples within 2 days.

The accuracy of the method was validated by measuring recovery through standard addition method. A known amount (low, medium and high) of the eighteen standard references were spiked into samples. Quantity of each component was subsequently obtained by use of the corresponding calibration plots. Each set of samples was analyzed three times. The RSD values were in the range of 1.17–4.78% and recoveries of analytes varied from 84.03% to 101.62%. Above results exhibited the reliability and accuracy for the measurement of these constituents. The recovery was calculated as follows: recovery (%) = 100 × (amount found − original amount)/amount spiked, as shown in Table S2.

### 2.3. Sample Analysis

**Figure 2:**
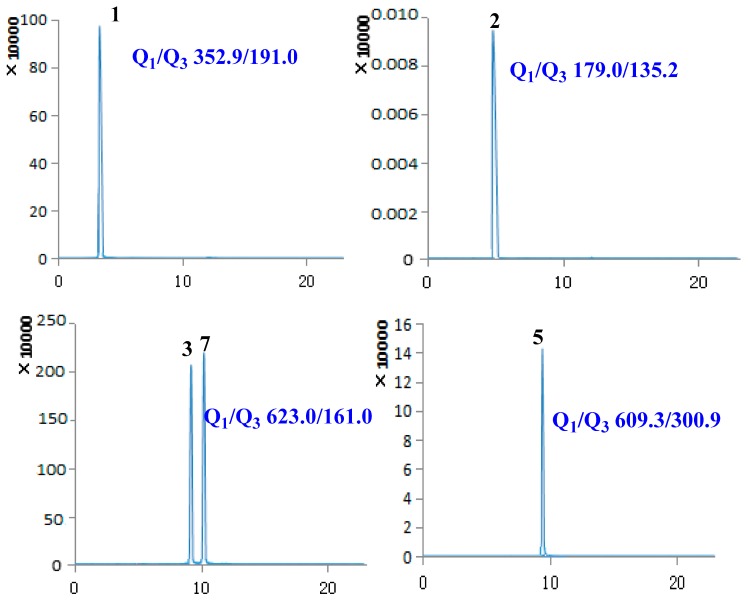

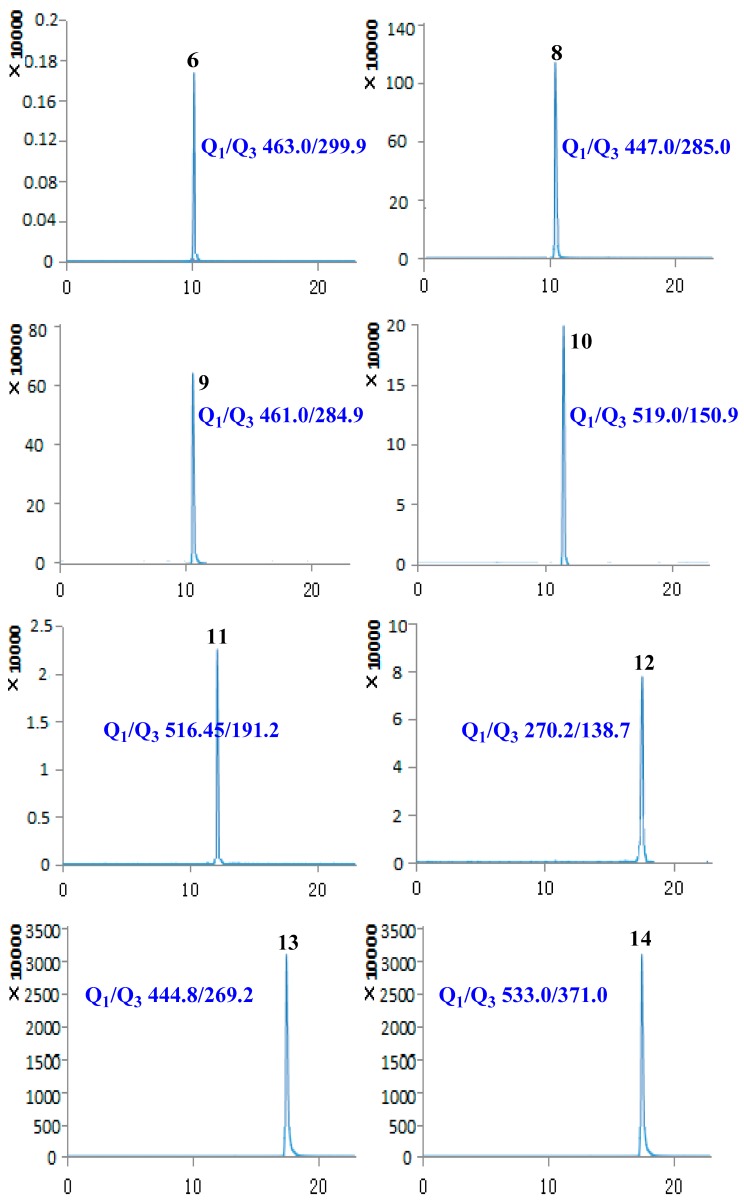

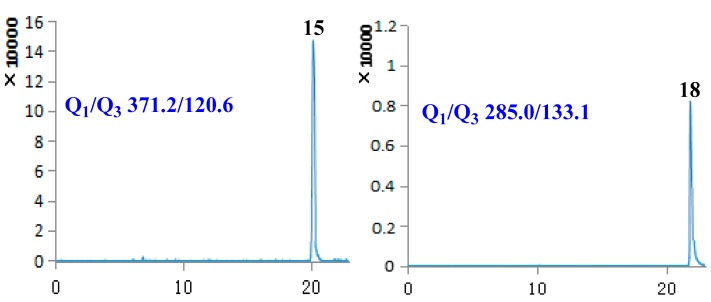
MRM chromatograms of **1**–**3**, **5**–**15** and **18** in sample.

The validated method was successfully applied for the identification and quantification of 18 active compounds in 14 batches of SHL oral liquids. Regarding as these 18 compounds due to multiple hydrophilic and lipophilic components from multiple classes, there were two quinic acids (**1** and **11**), one phenylpropionic acid (**2**), three phenylethanoid glycosides (**3**, **4** and **7**), eight flavonoids (**5**, **6**, **8**, **9**, **12**, **13**, **17** and **18**), four lignans (**10** and **14**–**16**). Meanwhile, these 18 constituents were classified into three groups according to their source of raw materials. It was characterized by eight active compounds **1**, **2**, **5**, **6**, **8**, **11**, **17** and **18** from Jinyinhua [12], four compounds **9**, **12**, **13** and **18** from Huangqin [13], and nine compounds **3**–**5**, **7**, **10** and **14**–**17** from Lianqiao [14]. Although three compounds **5**, **17** and **18** were simultaneously observed in two raw materials, other fifteen ingredients covered major and specific ones from different species. Also, this is the first report on simultaneous determination of these hydrophilic and lipophilic components in SHL oral liquid formulations by single LC-MS/MS run based on MRM mode. The contents of the investigated compounds (Figure 2), based on their respective calibration curves, are summarized in Table 3. Among these compounds, baicain (**13**) was found to be the most dominant constituents in all samples tested, at amounts of 12.27–15.90 mg/g. In addition, chlorogenic acid (**1**) and forsythiaside A (**7**) were also very abundant in 14 batches of SHL oral liquid. On the contrary, three compounds, forsythiaside B (**4**), arctiin (**16**) and quercetin (**17**), were not detected at all. This may be explained by specific processing method of SHL oral liquids.

**Figure 3:**
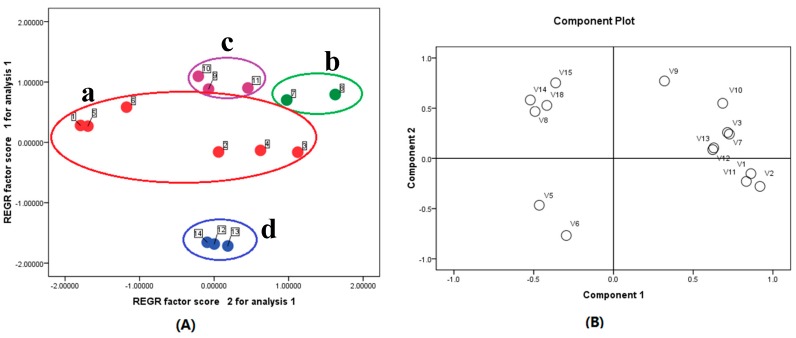
(**A**) Score plots from PCA; (**B**) loading plots from PCA.

Obvious differences could be further observed through performing principal component analysis (PCA) in Figure 3. PCA is a useful tool of chemometrician for data compression and information extraction which find combinations of variables or factors that describe major trends in a data set. It was noticeable that these 14 samples tested apparently form into four groups a–d according to their manufacturers (Figure 3A). The groups a–d were assigned to be Sanjing, Tailong, Zhenbaodao and Baitian’e, respectively. The corresponding PCA loading plot is illustrated in Figure 3B. Clearly, chlorogenic acid (**1**), lianqiaoxinside A (**3**), (+)-pinoresinol-β-d-glucoside (**10**), and phillygenin (**15**) were found at higher amounts in samples 7 and 8 from Tailong but caffeic acid (**2**) was lower in samples 7 and 8 compared with samples 9, 10 and 11 from Zhenbaodao. Thus, the concentration of some analytes varied greatly among the different samples, which was probably due to growing condition, climate, and drug processing of crude herbs. So, detection of a single or only several components could not effectively guarantee the quality of SHL oral liquids. It is essential for carry out simultaneous determination of multiple gradients for quality control of the herbal prescription.

## 3. Materials and Methods

### 3.1. Chemicals and Materials

Lianqiaoxinside A was isolated by the author from the fruits of *F. suspensa*. Chlorogenicacid, caffeic acid, forsythiaside B, rutin, hyperoside, forsythiaside A, cynaroside, scutellarin, (+)-pinoresinol-β-d-glucoside, isochlorogenic acid A, baicalein, baicalin, phillyrin, phillygenin, arctiin, quercetin, luteolin (≥98.0%)were purchased from the Chengdu MUST Biotechnology Co., Ltd. (Chengdu, China). HPLC grade acetonitrile (ACN) and formic acid were obtained from Fisher Scientific (Waltham, MA, USA). Purified water was used from a Milli-Q system (Millipore, Bedford, MA, USA). All other reagents were of analytical grade.

### 3.2. Preparation of Standard Solutions

The standards for Chlorogenic acid, caffeic acid, lianqiaoxinside A, forsythiaside B, rutin, hyperoside, forsythiaside A, cynaroside, scutellarin, (+)-pinoresinol-β-d-glucoside, isochlorogenic acid A, baicalein, baicalin, phillyrin, phillygenin, arctiin, quercetin, luteolin were weighed accurately and dissolved in methanol at a concentration of 1 mg mL^−1^. A mixed intermediate stock standard solution was then prepared in methanol; the concentrations of compounds in this solution were 10 ug·mL^−1^ except that baicalin was 40 ug·mL^−1^. The stock solutionsforeach quantitative analytes were further diluted with methanol to achieve a series of working solutions used to establish the calibration curves. The standard stock solutions and the working standard solutions were stored in brown vials at 4 °C.

### 3.3. Samples Preparation

14 batches of SHL oral liquid were collected from different manufacturers. Commercial product SHL 1–6 (lot No. 13112121, 13050633, 13050643, 13050653, 13031716, 13031816), SHL 7–8 (lot No. 131221042, 140111102), SHL 9–11 (lot No. B20130323, B20130108, B20130415) and SHL 12–14 (lot No. 130510, 130617, 130626) were purchased from Sanjing Pharmaceutical Co., Ltd. (Harbin, China), Tailong Pharmaceutical Co., Ltd. (Zhengzhou, China), Zhenbaodao Pharmaceutical Co., Ltd. (Harbin, China) and Baitian’e Pharmaceutical Co., Ltd. (Harbin, China), respectively. All sample solutions were ultrasonically extracted with methanol—water (50:50, *v*/*v*) for 20min. The sample solutions were filtered through a 0.22 μm membrane filter before it was injected into the UPLC system for analysis.

### 3.4. Chromatographic and MS Conditions

Analysis was performed using an ACQUITY UPLC system with a conditioned autosampler at 4 °C. Chromatographic separation was carried out at 35 °C on an ACQUITY UPLC HSS T3 column (1.8 μm, 2.1 mm × 100 mm). The mobile phase was composed of A (0.1% formic acid in water) and B (0.1% formic acid in acetonitrile) with a gradient elution: 0–5 min, 87–84% (A); 5–7 min, 84–80% (A); 7–15 min, 80–78% (A) and 15–23 min, 78–70% (A). The flow rate of the mobile phase was 0.2 mL/min, and the injection volume was 2 µL.

The mass spectrometry was performed on a 4000 QTRAP LC-MS/MS system (AB Sciex, Foster City, CA, USA) equipped with ESI interface in negative mode. All instruments were controlled and synchronized by Analyst software (version 1.6, SCIEX, AB Sciex, Foster City, CA, USA). Ion spray voltage was set at (3300) V, turbo spray temperature was 550 °C and interface heater was on. Both nebulizer gas (gas 1) and heater gas (gas 2) were set at 55 psi.

## 4. Conclusions

In this study, a new UPLC-ESI^−^-MRM-MS/MS method has been developed for the simultaneous determination of 18 major components in SHL oral liquid. This method was advantaged for rapid and simultaneous determination of multiple classes of hydrophilic and lipophilic components in SHL oral liquids by comparison with previous reports. These compounds include two quinic acids (**1** and **11**), one phenylpropionic acid (**2**), three phenylethanoid glycosides (**3**, **4** and **7**), eight flavonoids (**5**, **6**, **8**, **9**, **12**, **13**, **17** and **18**), four lignans (**10** and **14**–**16**). This novel evaluation approach can overcome the deficiencies of previously described methods revealing the complexity of samples from the same or different manufacturers. It provides much more qualitative information than any other singular evaluation. Data analysis on the 14 SHL oral liquid samples suggested that the concentration of the some compounds varied significantly from different manufacturers of China. The proposed method had been elucidated to be a simple, sensitive, accurate and reliable quality control procedure of SHL oral liquids.

## Figures and Tables

**Table 1:** Main MS parameters for UPLC-MS/MS analysis.

No.	Compound Name	Retention Time (min)	Molecular Weight	Selected Ion (*m*/*z*)	Q_1_	Q_3_	DP	CE
**1**	Chlorogenic acid	3.40	354.31	[M − H]^−^	352.90	191.00	−59.56	−19.70
**2**	Caffeic acid	5.02	180.15	[M − H]^−^	179.00	135.20	−60.54	−20.09
**3**	Lianqiaoxinside A	9.09	624.59	[M − H]^−^	623.00	161.00	−157.08	−57.85
**4**	Forsythiaside B	9.49	756.70	[M − H]^−^	755.00	593.60	−160.00	−50.93
**5**	Rutin	9.61	610.51	[M − H]^−^	609.30	300.90	−163.89	−47.96
**6**	Hyperoside	9.94	464.38	[M − H]^−^	463.00	299.90	−114.35	−36.97
**7**	Forsythiaside A	10.05	624.59	[M − H]^−^	623.00	161.00	−157.08	−57.85
**8**	Cynaroside	10.14	448.38	[M − H]^−^	447.00	285.00	−138.97	−39.83
**9**	Scutellarin	10.19	462.37	[M − H]^−^	461.00	284.90	−78.92	−26.24
**10**	(+)-Pinoresinol-β-d-glucoside	11.57	520.53	[M − H]^−^	519.00	150.90	−84.64	−44.99
**11**	Isochlorogenic acid A	12.09	516.45	[M − H]^−^	515.00	191.20	−75.30	−44.90
**12**	Baicalein	17.47	270.24	[M − H]^−^	269.00	138.70	−100.31	−42.71
**13**	Baicalin	17.50	446.37	[M − H]^−^	444.80	269.20	−89.57	−26.28
**14**	Phillyrin	20.13	534.56	[M − H]^−^	533.00	371.00	−65.42	−18.09
**15**	Phillygenin	20.14	372.41	[M − H]^−^	371.20	120.60	−94.80	−43.28
**16**	Arctiin	21.14	534.55	[M − H]^−^	533.00	371.20	−69.52	−20.93
**17**	Quercetin	21.89	302.24	[M − H]^−^	301.10	151.20	−88.89	−30.26
**18**	Luteolin	21.91	286.24	[M − H]^−^	285.00	133.10	−106.49	−47.79

**Table 2:** Summarization of calibration results, LOD and LOQ values.

No.	Regression Equation	Linear Range (μg/mL)	*R*^2^	LODs (ng/mL)	LOQs (ng/mL)
**1**	*y* = 4 × 10^6^*x* – 32,641	0.02–5.00	0.9993	4.88	9.76
**2**	*y* = 4 × 10^6^*x* + 52,653	0.02–5.00	0.9990	4.88	9.76
**3**	*y* = 1 × 10^6^*x* – 63,209	0.02–5.00	0.9992	4.88	9.76
**4**	*y* = 2482.3*x* − 626.91	0.31–10.00	0.9997	78.13	156.25
**5**	*y* = 375,597*x* – 14,725	0.02–2.50	0.9992	4.88	9.76
**6**	*y* = 78,413*x* − 2751.8	0.02–5.00	0.9999	9.76	19.53
**7**	*y* = 1 × 10^6^*x* – 88,370	0.02–10.00	0.9992	4.88	9.76
**8**	*y* = 604,060*x*+9178.1	0.04–10.00	0.9999	2.44	4.88
**9**	*y* = 3 × 10^6^*x* – 38,112	0.02–5.00	0.9995	4.88	9.76
**10**	*y* = 708,705*x* – 58,709	0.02–10.00	0.9993	2.44	4.88
**11**	*y* = 38,443*x* − 1932.4	0.02–5.00	0.9995	4.88	19.53
**12**	*y* = 13,450*x* + 85.951	0.08–10.00	0.9999	4.88	19.53
**13**	*y* = 1 × 10^6^*x* + 524,990	0.63–50.00	0.9991	19.53	78.13
**14**	*y* = 10,777*x* − 216.5	0.04–10.00	0.9997	2.44	9.77
**15**	*y* = 13,664*x* − 918.63	0.08–10.00	0.9998	9.77	39.06
**16**	*y* = 21,338*x* − 1861.1	0.04–10.00	0.9995	9.77	39.06
**17**	*y* = 1 × 10^6^*x* + 5883.7	0.02–10.00	0.9993	2.44	9.77
**18**	*y* = 1 × 10^6^*x* − 3265.6	0.02–10.00	0.9997	2.44	9.77

*y* is the peak areas of reference standards, and *x* is the value of the reference compound’s concentration (μg/mL).

**Table 3:** Contents (mg/g) of main constituents in 14 different batches of SHL oral liquids.

Samples	Manufacturers	Batches	1	2	3	4	5	6	7	8	9	10	11	12	13	14	15	16	17	18
Sample 1	Sanjing, Harbin	13112121	1.11	0.14	0.31	0	0.23	0.03	1.16	1.07	0.09	0.19	0.16	2.85	13.16	0.51	0.63	0	0	0.002
Sample 2	Sanjing, Harbin	13050633	0.91	0.17	0.51	0	0.15	0.02	2.04	1.51	0.12	0.34	0.14	2.98	12.63	0.43	0.65	0	0	0.002
Sample 3	Sanjing, Harbin	13050643	1.12	0.19	0.61	0	0.17	0.02	2.46	1.68	0.13	0.39	0.15	3.51	15.90	0.62	0.75	0	0	0.003
Sample 4	Sanjing, Harbin	13050653	1.14	0.22	0.56	0	0.16	0.02	2.14	1.62	0.13	0.37	0.13	2.91	14.63	0.58	0.71	0	0	0.002
Sample 5	Sanjing, Harbin	13031716	1.05	0.16	0.37	0	0.23	0.03	1.52	0.26	0.10	0.23	0.11	2.56	12.85	0.57	0.69	0	0	0.001
Sample 6	Sanjing, Harbin	13031816	1.23	0.18	0.36	0	0.24	0.03	1.42	0.27	0.12	0.23	0.13	2.99	14.39	0.60	0.67	0	0	0.001
Sample 7	Tailong, Henan	131221042	1.28	0.32	0.88	0	0.13	0.02	3.28	0.03	0.12	0.49	0.28	2.95	13.59	0.61	0.81	0	0	0.001
Sample 8	Tailong, Henan	140111102	1.41	0.36	0.83	0	0.11	0.02	3.21	0.03	0.15	0.50	0.35	2.96	14.07	0.58	0.84	0	0	0.001
Sample 9	Zhenbaodao, Harbin	B20130323	1.15	0.44	0.30	0	0.12	0.02	1.30	0.02	0.12	0.27	0.27	3.03	14.41	0.50	0.63	0	0	0.001
Sample 10	Zhenbaodao, Harbin	B20130108	1.18	0.38	0.10	0	0.09	0.02	0.41	0.04	0.12	0.22	0.46	3.34	13.76	0.44	0.59	0	0	0.001
Sample 11	Zhenbaodao, Harbin	B20130415	1.16	0.41	0.36	0	0.12	0.02	1.45	0.02	0.14	0.29	0.28	3.32	15.00	0.52	0.63	0	0	0.001
Sample 12	Baitianer, Harbin	130510	0.89	0.004	0.04	0	0.16	0.02	0.08	1.14	0.13	0.22	0.05	2.65	12.29	0.77	0.95	0	0	0.002
Sample 13	Baitianer, Harbin	130615	0.86	0.002	0.03	0	0.14	0.02	0.06	1.11	0.14	0.22	0.03	2.67	12.27	0.74	0.93	0	0	0.002
Sample 14	Baitianer, Harbin	130311	0.88	0.004	0.02	0	0.15	0.02	0.07	1.13	0.12	0.23	0.06	2.64	12.30	0.77	0.96	0	0	0.002

## Supplementary Materials

Supplementary Materials are available online.
