# Supplementary material for: Simultaneous Determination of Multiple Classes of Hydrophilic and Lipophilic Components in Shuang-Huang-Lian Oral Liquid Formulations by UPLC-Triple Quadrupole Linear Ion Trap Mass Spectrometry
Source: Molecules. 2017 Nov 24;22(12):2057. doi: 10.3390/molecules22122057 (PMC6149676; doi:10.3390/molecules22122057)
Supplement: Supplementary file 1 [file molecules-22-02057-s001.pdf]

Table S1. Intra- and inter-day variability for the assay of the 18 constituents

No.	Concentrations (μg/mL)	Intra-day Found	Intra-day RSD (%)	Intra-day Accuracy (%)	Inter-day Found	Inter-day RSD (%)	Inter-day Accuracy (%)
**1**	2.50	2.61±0.03	1.15	104.40	2.47±0.06	2.43	98.80
	1.25	1.28±0.01	0.78	102.40	1.19±0.02	1.28	95.47
	1.25	1.21±0.02	1.65	96.80	1.20±0.03	2.50	96.00
	0.63	0.66±0.01	1.52	104.76	0.61±0.02	3.28	96.83
**2**	2.50	2.59±0.03	1.16	103.60	2.48±0.02	0.81	99.20
	1.25	1.19±0.02	1.28	95.47	1.30±0.01	0.45	103.73
	1.25	1.28±0.03	2.34	102.40	1.22±0.04	3.28	97.60
	0.63	0.60±0.01	1.67	95.24	0.66±0.01	1.52	104.76
**3**	2.50	2.55±0.07	2.75	102.00	2.49±0.03	1.20	99.60
	1.25	1.30±0.03	2.48	103.73	1.20±0.02	1.27	96.27
	1.25	1.22±0.01	2.34	102.40	1.20±0.03	2.50	96.00
	0.63	0.60±0.01	1.67	95.24	0.64±0.01	1.56	101.59
**4**	2.50	2.58±0.04	1.55	103.20	2.62±0.02	0.76	104.80
	1.25	1.30±0.03	1.93	104.27	1.29±0.02	1.55	103.20
	1.25	1.20±0.03	2.50	96.00	1.28±0.02	1.56	102.40
	0.63	0.62±0.02	3.23	98.41	0.65±0.01	1.54	103.17
**5**	2.50	2.44±0.04	1.64	97.60	2.56±0.06	2.34	102.40
	1.25	1.19±0.02	1.28	95.47	1.20±0.02	1.28	95.73
	1.25	1.27±0.03	2.36	101.60	1.29±0.04	3.10	103.20
	0.63	0.66±0.01	1.52	104.76	0.65±0.02	3.08	103.17
**6**	2.50	2.57±0.05	1.95	102.80	2.58±0.04	1.55	103.20
	1.25	1.31±0.01	0.76	104.80	1.27±0.03	2.36	101.60
	0.63	0.63±0.02	3.17	100.00	0.61±0.02	3.28	96.83
**7**	2.50	2.48±0.03	1.21	99.20	2.55±0.04	1.57	102.00
	1.25	1.19±0.02	1.68	95.20	1.22±0.03	2.46	97.60
	0.63	0.61±0.02	3.28	96.83	0.62±0.02	3.23	98.41
**8**	2.50	2.52±0.04	1.59	100.80	2.57±0.05	1.95	102.80
	1.25	1.19±0.03	2.52	95.20	1.23±0.03	2.44	98.40
	0.63	0.63±0.01	1.59	100.00	0.61±0.01	1.64	96.83
**9**	2.50	2.45±0.02	0.82	98.00	2.54±0.02	0.79	101.60
	1.25	1.26±0.04	3.17	101.80	1.31±0.01	0.76	104.80
	0.63	0.60±0.02	3.33	95.24	0.64±0.01	1.56	101.59
**10**	2.50	2.43±0.05	2.06	97.20	2.39±0.01	0.42	95.60
	1.25	1.20±0.02	1.67	96.00	1.25±0.03	2.40	100.00
	0.63	0.62±0.02	3.23	98.41	0.61±0.01	1.64	96.83
**11**	2.50	2.57±0.04	1.56	102.80	2.60±0.03	1.15	104.00
	1.25	1.30±0.02	1.54	104.00	1.28±0.02	1.56	102.40
	0.63	0.62±0.01	1.61	98.41	0.60±0.02	3.33	95.24
**12**	2.50	2.51±0.04	1.59	100.40	2.50±0.01	0.40	100.00
	1.25	1.27±0.04	3.15	101.60	1.26±0.02	1.59	100.80
	0.63	0.66±0.02	3.03	104.76	0.63±0.02	3.17	100.00
**13**	10.00	9.91±0.07	0.71	99.10	10.12±0.09	0.89	101.20
	5.00	5.12±0.08	1.56	102.40	5.09±0.06	1.18	101.80
	2.50	2.49±0.04	1.61	99.60	2.53±0.05	1.98	101.20
**14**	2.50	2.42±0.03	1.24	96.80	2.52±0.03	1.19	100.80
	1.25	1.26±0.04	3.17	100.80	1.20±0.02	1.67	96.00
	0.63	0.62±0.02	3.23	98.41	0.63±0.02	3.17	100.00
**15**	2.50	2.56±0.06	2.34	102.40	2.48±0.04	1.61	99.20
	1.25	1.19±0.03	2.52	95.20	1.23±0.02	1.63	98.40
	0.63	0.67±0.02	2.99	106.35	0.65±0.02	3.08	103.17
**16**	2.50	2.44±0.05	2.05	97.60	2.53±0.07	2.77	101.20
	1.25	1.29±0.03	2.33	103.20	1.30±0.03	2.31	104.00
	0.63	0.61±0.02	3.28	96.83	0.63±0.02	3.17	100.00

**17**	2.50	2.48 ± 0.04	1.61	99.20	2.41 ± 0.05	2.07	96.40
	1.25	1.29 ± 0.02	1.55	103.20	1.30 ± 0.02	1.54	104.00
	0.63	0.62 ± 0.02	3.23	98.41	0.65 ± 0.02	3.08	103.17
**18**	2.50	2.60 ± 0.02	0.77	104.00	2.54 ± 0.04	1.57	101.60
	1.25	1.22 ± 0.02	1.64	97.60	1.26 ± 0.04	3.17	100.80
	0.63	0.61 ± 0.02	3.28	96.83	0.62 ± 0.02	3.23	98.41

[a] RSD (%) = (SD /mean) × 100

[b] Accuracy (%) = Found/original concentration × 100

Table S2. Recovery results of compounds tested

No.	Original (mg/g)	Spiked (mg)	Found (mg)	Mean recovery (%)	Average (%)	RSD (%) (n = 3)
**1**	1.12	0.57	1.63	89.47	92.10	2.52
		1.14	2.19	93.86		
		1.71	2.71	92.98		
**2**	0.19	0.09	0.27	88.89	85.80	3.30
		0.18	0.34	83.33		
		0.27	0.42	85.19		
**3**	0.61	0.32	0.92	96.88	94.45	2.30
		0.64	1.21	93.75		
		0.96	1.50	92.71		
**5**	0.17	0.09	0.25	88.89	91.97	3.07
		0.18	0.34	94.44		
		0.27	0.42	92.59		
**6**	0.02	0.01	0.0296	96.00	95.94	1.48
		0.02	0.0389	94.50		
		0.03	0.0492	97.33		
**7**	2.46	1.27	3.76	102.36	100.62	2.01
		2.52	4.94	98.41		
		3.65	6.15	101.10		
**8**	1.68	0.82	2.40	87.80	89.22	1.42
		1.64	3.15	89.63		
		2.46	3.90	90.24		
**9**	0.13	0.07	0.19	85.71	89.68	4.06
		0.14	0.26	92.86		
		0.21	0.32	90.48		
**10**	0.39	0.19	0.57	94.74	97.95	3.62
		0.38	0.76	97.37		
		0.57	0.97	101.75		
**11**	0.15	0.08	0.22	87.50	84.03	3.79
		0.16	0.28	81.25		
		0.24	0.35	83.33		
**12**	3.51	1.81	5.30	98.90	100.24	1.27
		3.49	7.05	101.43		
		5.32	8.85	100.38		
**13**	15.90	8.07	24.14	102.11	101.62	1.26
		15.84	32.15	102.59		
		23.72	39.66	100.17		
**14**	0.62	0.34	0.97	102.94	101.14	1.96
		0.68	1.31	101.47		

		1.02	1.63	99.02		
**15**	0.75	0.37	1.11	97.30	97.15	1.17
		0.74	1.46	95.95		
		1.11	1.84	98.20		
**18**	0.003	0.0018	0.0045	83.33	87.35	4.78
		0.0036	0.0063	91.67		
		0.0054	0.0077	87.04		
